# Gastrointestinal microbiota and barrier integrity in individuals who develop exertional heat illness and pair‐matched controls: A prospective observational cohort study

**DOI:** 10.1113/EP093100

**Published:** 2026-05-25

**Authors:** Alex A. M. Gould, Neil P. Walsh, Michael J. Tipton, Michael J. Zurawlew, Samuel C. Robson, Janis K. Shute, Joy E. M. Watts, Hayley C. Tyson, Megan R. Robinson, Andrew J. Roberts, Alex J. Rawcliffe, Ross Hemingway, Jo Corbett

**Affiliations:** ^1^ Extreme Environments Laboratory, School of Psychology, Sport, and Health Sciences, Faculty of Science and Health University of Portsmouth Portsmouth UK; ^2^ School of Sport and Exercise Sciences Liverpool John Moores University Liverpool UK; ^3^ Institute of Life Sciences and Healthcare & School of Medicine, Pharmacy and Biomedical Sciences, Faculty of Science and Health University of Portsmouth Portsmouth UK; ^4^ Institute of the Earth and Environment School of the Environment and Life Sciences University of Portsmouth Portsmouth UK; ^5^ Army Recruit Health and Performance Research, Medical Branch, HQ Army Individual Training Command Ministry of Defence Upavon UK; ^6^ Medical Centre Commando Training Centre Royal Marines Lympstone UK

**Keywords:** exercise induced gastrointestinal syndrome, heat stroke, microbiome

## Abstract

It has been hypothesised that the composition of the gastrointestinal (GI) microbiota contributes to exertional heat illness (EHI) aetiology, but relevant empirical data in humans are lacking. Utilising a unique *prospective* study design, stool samples and resting blood samples were obtained from 550 individuals *prior to* (within 3 days) undertaking a 6.4‐mile/10.3 km loaded march (median (IQR) duration = 66 (1) min), during which 79 individuals developed an EHI (mild, *n *= 55; severe, *n *= 24). These individuals were pair‐matched for body mass index and cardiorespiratory fitness to individuals who did not develop an EHI during the same exercise (non‐EHI). Our primary outcome measure was the composition of the gut microbiota, determined using 16S ribosomal RNA (rRNA) amplicon sequencing of stool samples. Secondary outcomes included the concentration of baseline blood biomarkers of GI barrier integrity (intestinal fatty acid binding protein, claudin 3, zonulin, lipopolysaccharide binding protein, and soluble cluster of differentiation 14). No significant differences in the composition of the GI microbiota (α‐diversity, β‐diversity, relative abundance, differential abundance) were observed between EHI cases and matched non‐EHI controls (*P *> 0.05). Similarly, no significant between‐group differences in biomarkers of GI barrier integrity were observed. These findings persisted when conducting additional sub‐group analysis of severe EHI cases only, and additional sensitivity analysis excluding individuals who reported non‐steroidal anti‐inflammatory drug use and/or GI disorders. In conclusion, when potential confounding factors are controlled for, the composition of the GI microbiota and baseline GI barrier integrity do not appear to predispose to increased EHI risk during strenuous exercise.

## Abstract (highlights)

- **What is the central question of this study?**
  Do the GI microbiota composition and indices of baseline GI barrier integrity differ between individuals who subsequently develop an EHI and matched control individuals who do not develop an EHI?
- **What is the main finding and its importance?**
  The GI microbiota composition and indices of GI barrier integrity did not differ between EHI cases and matched non‐EHI controls. For this cohort and experimental model, when potential confounding variables are controlled for, neither the GI microbiota composition nor indices of GI barrier integrity contributes to a heightened risk of developing EHI during strenuous physical activity.

## 1 INTRODUCTION

Exertional heat illnesses (EHIs) encompass a range of conditions varying in severity from mild symptoms (e.g., headache, dizziness and nausea) to more severe symptoms including central nervous system (CNS) dysfunction and end‐organ damage (Hemingway et al., 2025; Laitano et al., 2019; Roberts et al., 2021). Severe EHIs may be fatal (Porter, 2000; Rav‐Acha et al., 2004) and, where an individual survives the initial event, are associated with adverse sequalae (Lawton et al., 2019; Tseng et al., 2020; Wang et al., 2019) and earlier all‐cause mortality (Wallace et al., 2007). Traditionally, EHIs have been viewed as arising from an imbalance between heat production and dissipation, which overwhelms the thermoregulatory system resulting in a dangerously elevated (e.g., >40°C) deep body temperature (Atha, 2013; Edwards et al., 1975; Roberts et al., 2021). However, athletes often attain deep‐body temperatures exceeding those associated with EHI development without incident (Byrne et al., 2006; Pugh et al., 1967; Racinais et al., 2019), whereas an individual may succumb to an EHI during exposure to a level of exercise heat‐stress that they have previously tolerated (Carter et al., 2007). Thus, it has been suggested that EHI aetiology is more complex than a thermoregulatory insufficiency, with a growing body of literature suggesting that the gastrointestinal (GI) system may be implicated in EHI aetiology (Armstrong et al., 2018; Garcia et al., 2022; Lim, 2018; Ogden et al., 2020).

Sustained exertional heat‐stress can cause GI ischaemia, damaging the GI epithelial barrier (van Wijck et al., 2011) and increasing the translocation of gram‐negative bacteria (e.g., lipopolysaccharide [LPS]) from the GI tract into the systemic circulation. Where this exceeds the neutralising ability of the liver, LPS binds to toll‐like receptor 4 on macrophages and lymphocytes (Fukui, 2016), triggering the acute phase response (Garcia et al., 2022; Leon, 2007), the production of pro‐inflammatory cytokines (e.g., tumour necrosis factor [TNF] α, interleukin [IL]‐6), and subsequently, anti‐inflammatory cytokines (e.g., IL‐1ra, IL‐10). When LPS accumulates in the systemic circulation, the resultant endotoxaemia can trigger the systemic inflammatory response (Garcia et al., 2022) that is implicated in the tissue and multi‐organ damage characterising the most severe forms of EHI (Carvalho et al., 2016; Li et al., 2022; Ubaldo et al., 2020).

The GI microbiota refers to the collection of microorganisms (bacteria, archaea, eukaryotes) contained within the GI tract, with their associated genomes termed the GI microbiome (Ursell et al., 2012). The gut microbiota exerts a wide range of effects on host physiology (Adak & Khan, 2019) including on factors potentially implicated in EHI aetiology, such as epithelial barrier homeostasis and integrity (Ghosh et al., 2021), inflammation (Scheithauer et al., 2020) and possibly temperature regulation (Bennett et al., 2020; Conn et al., 1991; Kluger et al., 1990). The composition and diversity of the gut microbiota may vary considerably between individuals (Falony et al., 2016), and also acutely within individuals due to multifarious factors including disease (Hou et al., 2022), pathogenic infection (Baümler & Sperandio, 2016; Rooks & Garrett, 2016), medication (Dethlefsen et al., 2008; Rogers & Aronoff, 2016), diet (David et al., 2014), environment (Gacesa et al., 2022) and exercise (Mohr et al., 2020; Monda et al., 2017), with multiple downstream consequences on functionality (Hou et al., 2022). Consequently, it has been hypothesised that the composition of the GI microbiota may have relevance in EHI aetiology and risk (Armstrong et al., 2012; Roberts et al., 2021). Additionally, GI hyperpermeability (Camilleri, 2019; Fasano, 2020) may increase the amount of LPS entering the portal circulation and has been putatively linked to the development of various diseases characterised by a pro‐inflammatory state (Chelakkot et al., 2018; Farhadi et al., 2003; Fasano, 2012; Simeonova et al., 2018). However, at present, relevant empirical studies examining the GI microbiota and barrier integrity in human EHI models are limited.

Previous research in healthy humans exposed to heat stress suggests that some bacterial groups are associated with the magnitude of GI barrier disturbances (e.g., Akkermansiaceae, *Ruminococcaceae*  family groups), inflammation (e.g., *Faecalibacterium*, *Ruminiclostridium‐9* genus groups) and thermoregulatory strain (e.g., *Acidaminococcaceae*, *Prevotellaceae*, *Ruminococcaceae* family groups) (Bennett et al., 2020), with the latter potentially due to pyrogenic effects relating to inflammatory cytokinaemia as a result of GI barrier disturbance and increased bacterial endotoxin translocation (Bennett et al., 2020). Thus, the relative abundance of certain bacterial groups within an individual's GI microbiota may influence several factors that are implicated in EHI aetiology. Similarly, physiological adaption to heat (i.e., acclimation) may decrease the abundance of pathogenic bacteria and increase probiotic bacteria that are hypothesised to counteract stress‐induced changes in intestinal barrier function and mucosal inflammation (Liu et al., 2023). In contrast, we have recently shown limited differences in either the composition of the GI microbiota or the GI barrier and inflammatory response to exercise heat‐stress in recent EHI patients compared to a matched group of individuals without prior EHI history (Gould et al., 2025). This suggests that a recent EHI episode does not result in significant ongoing abnormality in the GI microbiota and GI barrier function, and is unlikely to contribute to previously reported elevated ongoing EHI risk in this cohort (Nelson et al., 2018a, 2018b; Stearns et al., 2020). However, given the potential for acute changes in GI microbiota (i.e., between the EHI incident and the post‐incident assessment), it does not preclude the possibility that the composition of the GI microbiota and associated effects on GI barrier integrity and inflammation may have contributed to the initial incident. To address this hypothesis, a unique *prospective* research design is required, whereby the GI microbiota and indices of GI barrier integrity and function are assessed in close proximity *before* an EHI incident.

Accordingly, the aim of the present study was to *prospectively* examine the GI microbiota composition and indices of GI barrier integrity in individuals who subsequently developed an EHI in comparison to matched non‐EHI controls, who did not develop an EHI during the same activity. We hypothesised that the composition of the GI microbiota and/or indices of GI barrier integrity of individuals who subsequently developed an EHI would differ from those of matched non‐EHI controls, who did not develop an EHI during the same activity.

## 2 METHODS

### 2.1 Ethical approval

The study protocol was approved by the Ministry of Defence Research Ethics Committee (Protocol number 2029/MODREC/21), was registered at www.clinicaltrials.org (NCT04979455), and was conducted in accordance with the *Declaration of Helsinki* (2013). All volunteers were fully briefed prior to obtaining their written informed consent.

### 2.2 Study design

This study formed part of a larger 4‐year prospective observational‐cohort study to identify risk factors for EHI development. However, for logistical reasons it was only possible to obtain stool samples for the microbiome analysis and data reported in the present study for the first 3 years of the data collection programme. For the microbiome analysis reported in the present study, and based upon the effect‐size for α‐diversity reported in our previous work (Simpson index, *d *= 0.59; Gould et al., 2025), with a two‐tailed type 1 error rate of 5% and 1:1 allocation ratio, our sample size provided 96% power to detect a between‐group difference between individuals who developed an EHI and matched non‐EHI controls who did not develop an EHI during the same activity (G*Power 3.1.9.4).

### 2.3 Participants

**FIGURE 1:**
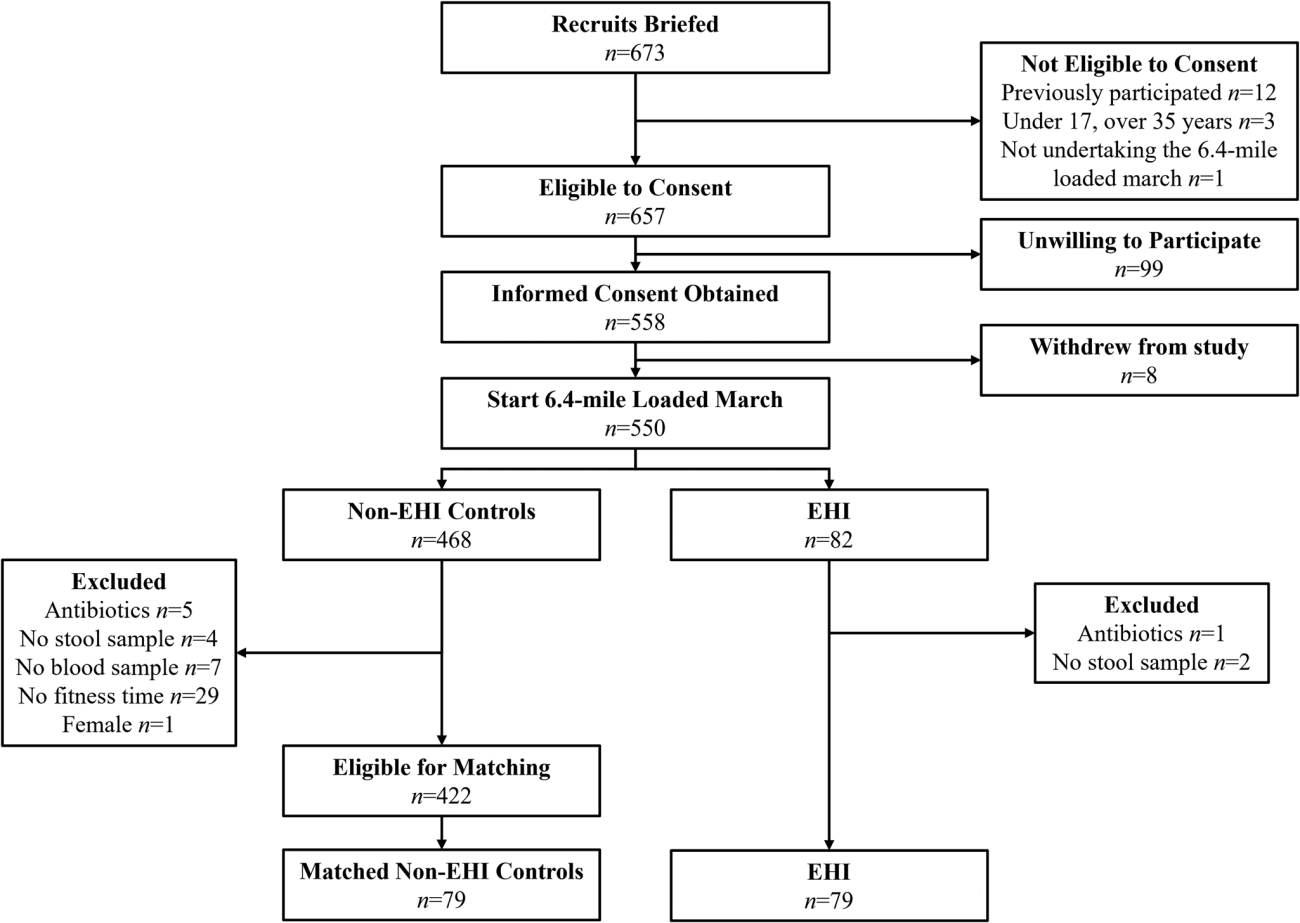
Consort flow diagram detailing the participant throughput during the study. EHI: exertional heat illness.

Between the months of March and October 2021–2023, 673 (672 = male; 1 = female) individuals were initially briefed, with 657 meeting the eligibility criteria. These included being aged 17 to 35 years of age, undertaking a 6.4‐mile/10.3 km loaded march in week 22 of Royal Marine Commando Training or week 7 of the All‐Arms Commando Course at Commando Training Centre RM (CTCRM), Devon, UK, and being free from cardiovascular, respiratory (e.g., asthma), digestive, kidney, neurological or endocrine disorders. Individuals reporting as pregnant were ineligible to participate (*n* = 0). From the eligible pool, 558 participants enrolled onto the study, with 550 starting the loaded march. Of those undertaking the loaded march, 82 participants (15%) met the a priori criteria for EHI, with three EHI cases excluded (details in Figure 1), leaving 79 EHI cases (55 mild; 24 severe). From a pool of 468 potential non‐EHI control participants, 46 met the additional exclusion criteria, leaving 422 non‐EHI controls eligible for subsequent pair matching (detailed subsequently). A consort flow diagram showing participant throughput is provided in Figure 1.

### 2.4 6.4‐mile loaded march

At ∼07.00 h, wet‐bulb globe temperature (WBGT) was recorded at the start and finish location of the loaded march (QUESTTemp 34, Quest Technologies Ltd, London, UK). Following a standardised warm‐up led by the training staff, participants commenced the loaded march. Participants wore a standard issue long‐sleeved T shirt, combat trousers and boots, and carried an external load and rifle totalling ∼14.5 kg. The loaded march was completed on a standardised undulating road route at ∼10‐minute mile pace. Participants marched or ran as a unit as directed by experienced physical training staff and, in line with local policy, a 1–2‐min ad libitum water stop was provided after ∼30 min. Upon cessation of exercise, each participant met with a member of the research team to report whether they were experiencing exercise‐induced symptoms of mild EHI and to complete the Quick Confusion Scale (QCS; Irons et al., 2002).

### 2.5 EHI case and non‐EHI control participant identification

Participants were classified as mild or severe EHI using key discriminatory features outlined in expert statements (Armstrong et al., 2007; Casa et al., 2015; Hemingway et al., 2025; Roberts et al., 2021). Mild EHI reported exercise‐induced symptoms of headache, dizziness or nausea during the loaded march (Roberts et al., 2021). Severe EHI exhibited: (i) CNS disturbance, the main diagnostic criterion for exertional heat stroke, determined as incapacitation (e.g., loss of consciousness) or a QCS score ≤11 (Irons et al., 2002); and (ii) hyperthermia determined as peak gastrointestinal temperature (*T*_gi_) ≥ 39.5°C (Gagnon et al., 2010) and/or, where referred for serial blood testing, evidence of end‐organ damage (Day 0 to Day +3). Acute kidney injury was determined as circulating creatinine ≥115 µmol L^−1^ (Fox et al., 2004; Khwaja, 2012) and/or acute liver injury as circulating alanine aminotransferase ≥89.5 U/L (Giannini et al., 2005; Prati et al., 2002). The remaining participants were classified as non‐EHI.

Additional exclusion criteria were applied prior to the non‐EHI control pair‐matching and analysis stage. Specifically, participants were excluded if they had consumed antibiotics within the last month (self‐reported by LimeSurvey) or were unable to provide a stool sample. Additionally, potential non‐EHI controls were removed from the pair matching pool if there was no baseline blood sample, body mass index (BMI) data or fitness time available. These two additional exclusion steps for non‐EHI controls were taken to ensure, as far as possible (i) complete data sets were available and (ii) EHI cases could be matched to an appropriate non‐EHI control for both BMI and fitness time, where these data were available for the EHI case (described subsequently). The only exception was for three course cohorts (troops) for whom, due to logistical constraints, we were unable to collect blood samples. However, these individuals were still included in our primary analysis of the GI microbiota. One female completed the 6.4‐mile loaded march without developing an EHI, but as no female EHI cases were identified, this individual was excluded from the pool of available non‐EHI controls for subsequent pair matching.

Whilst non‐steroidal anti‐inflammatory drug (NSAID) use (Rogers & Aronoff, 2016) and GI disorders (Michielan & D'Incà, 2015) may affect the GI microbiota, we elected not to exclude NSAID users and individuals reporting a GI disorder (either as cases or non‐EHI controls) from our participant pool due to: (i) high reported NSAID usage rates amongst this cohort; (ii) the potential for these factors to affect EHI risk via their influence on the GI microbiota; and (iii) desire to ensure a research approach with strong ecological validity in the study population. However, acknowledging these potential confounders, we also undertook sensitivity analysis excluding NSAID users and individuals with GI disorders (i.e., if either a ‘case’ or ‘control’ reported NSAID use or GI disorder, the pair was excluded from analysis), as detailed subsequently.

### 2.6 Non‐EHI control pair‐matching

Each course cohort had been undertaking identical training as part of their course prior to the 6.4‐mile loaded march, eating similar food and drink whilst on base, or out on exercise (i.e., standard meals from mess or ration packs), and living in the same accommodation. Therefore, for each identified EHI ‘case’, the corresponding non‐EHI ‘control’ was selected from the eligible pool of potential non‐EHI control participants within the same course cohort, thus controlling for factors that may influence the GI microbiota, including diet (Singh et al., 2017), seasonal differences (Gacesa et al., 2022) and physical activity levels (Monda et al., 2017), as well as ambient conditions during the loaded march (Jenkins et al., 2023). Two other variables that can confound microbiome analysis are BMI (Vujkovic‐Cvijin et al., 2020) and cardiovascular fitness (Estaki et al., 2016). To control for these factors, we used the Euclidean distance method approach, similar to that employed by Vujkovic‐Cvijin et al. (2020), to identify the participant within each course cohort that was most closely matched to the EHI case for both BMI and fitness.

It was not possible to obtain data for all participants from a single common fitness test. As such, two fitness tests were used as an index of cardiorespiratory fitness: (i) Royal Marines Fitness Test (RMFT), and (ii) Bottom Field Test (BFT). The RMFT consists of a ‘best effort’ 2.5‐miles/4.0 km loaded march carrying webbing (∼9.5 kg), daysack (∼11 kg) and rifle (∼5 kg). The BFT consists of an assault course of 12 obstacles which individuals must complete in <5 min to pass. RMFT times were used as the primary index of fitness, with BFT time used where this was not available. For one course cohort, only the RMFT finishing order was available; these data were grouped into rank quartiles as the fitness index. If an identified EHI case did not have a fitness measure (*n *= 9), they were matched to the non‐EHI controls solely on BMI. A script was produced in R v4.2.3 (R Core Team, 2023) to automate the selection of BMI‐ and fitness‐matched non‐EHI controls for each course cohort. This used a *Z*‐score normalisation approach to standardise BMI and fitness scores, for each individual within their respective course cohort. Next, the script identified the eligible non‐EHI control participant within the course cohort with the smallest Euclidean distance for each EHI case. In instances where the same non‐EHI control was identified for multiple EHI cases within the cohort, the non‐EHI control was allocated to the EHI case with smallest Euclidean distance and, for the remaining case, the non‐EHI control with the next smallest Euclidean distance was selected. This was done automatically for *n *= 2, and manually for the rare occasions where *n *> 2.

### 2.7 General procedures

A single stool sample was provided by each participant in the 3 days preceding the 6.4‐mile loaded march, free from urine, using a sterile collection kit (80.734.001, Sarstedt, Nümbrecht, Germany). Upon receipt, stool samples were placed on ice and immediately taken and placed into a −80°C freezer until transportation on dry ice to the University of Portsmouth for DNA extraction. Extracted DNA was subsequently transported on dry ice to the University of Southampton for 16S rRNA amplicon sequencing (see below). On the morning 1 day prior to the loaded march, height (HM‐250P, Marsden, Rotherham, UK), and body mass (Seca 704, Seca, Germany, Hamburg) were obtained, with body surface area subsequently calculated using the Du Bois and Du Bois equation (Du Bois & Du Bois, 1916). In addition, 12 mL of venous blood was taken from the antecubital vein obtained in a seated position using a 21‐gauge needle (Becton Dickinson, Franklyn Lakes, NJ, USA) and collected into a serum (6 mL; Becton Dickinson) and EDTA (6 mL; Becton Dickinson) vacutainer and inverted 6–8 times. A centrifuge (5810R, Eppendorf, Hamburg, Germany) was used (1500 *g* for 10 min at 4°C) to separate the plasma from the cells in the remainder of the sample. Serum vacutainers were left to clot for 1 h before being centrifuged. Aliquots of plasma and serum were stored in microcentrifuge tubes and frozen at −80°C for subsequent biochemical analyses. On the evening preceding the loaded march, participants ingested a GI telemetry capsule (BodyCap, Caen, France; Cortemp, HQ inc, Palmetto, FL, USA). Prior to ingestion, each telemetry capsule underwent a three‐point calibration (at 36, 39 and 42°C) to ensure error was within ±0.1°C of a certified and calibrated thermometer (Spirit ASTM, Cumbria, Cleator Moor, UK). Sampling epoch was set to 10 s and was subsequently averaged over each minute of the 6.4‐mile loaded march. Erroneous *T*_gi_ data (>0.3°C difference between consecutive 10 s samples) were removed prior to analysis.

### 2.8 Bacterial DNA extraction

Microbial DNA was extracted from stool samples in batches with a positive (D6300, ZymoBIOMICS Microbial Community Standard, Irvine, CA, USA) and negative control (nuclease free water). Bacterial DNA was extracted using the QIAamp PowerFecal Pro DNA kit (51804, Qiagen, Hilden, Germany) according to the manufacturer's instructions. DNA was quantified by a spectrophotometer (DS‐11, DeNovix, Wilmington, DE, USA). The purified DNA was stored at −80°C prior to sequencing.

### 2.9 16S rRNA gene sequencing

Purified DNA was used for 16S rRNA gene amplicon sequencing. A PCR workflow was performed for library preparation using universal primers specific for the V3 and V4 hypervariable regions of the 16S rRNA gene (341F 5′‐CCTACGGGNGGCWGCAG‐3′ and 805R 5′‐GACTACHVGGGTATCTAATCC‐3; Klindworth et al., 2013). PCR reactions were conducted on an Applied Biosystems Veriti 96 Well Thermal Cycler (Thermo Fisher Scientific, Waltham, MA, USA) using the following protocol: (i) initial denaturation for 3 min at 98°C; (ii) 25 cycles of denaturation for 30 s at 98°C, annealing for 30 s at 55°C, extension for 30 s at 72°C; (iii) final extension for 5 min at 72°C. AMPure XP beads (Beckman Coulter, Brea, CA, USA) were used to purify the 16S rRNA gene V3 and V4 amplicons. Individual libraries were indexed with 8 cycles of PCR using Illumina (San Diego, CA, USA) DNA/RNA UD indexes and purified again using AMPure XP beads. Individual libraries were quantified using the Quant‐iT dsDNA High‐Sensitivity Assay Kit (Thermo Fisher Scientific) and quality controlled using the Agilent Bioanalyzer DNA1000 kit (Agilent Technologies, Santa Clara, CA, USA), prior to normalisation and pooling. Cluster generation and sequencing were conducted on an Illumina MiSeq benchtop sequencer using an Illumina MiSeq Reagent Kit v3 (600‐cycle).

### 2.10 Bioinformatics

*FastQC* v0.11.9 (Andrews, 2012) was used to assess the quality of the raw read data to ensure the absence of sequencing artefacts. Reads were trimmed to remove adapter sequences and poor quality reads using *trim_galore* v0.6.7 (Krueger, 2012) using parameters ‘‐q 20 –stringency 5 ‐e 0.1 –length 20 –trim‐n –clip_R1 10 –clip_R2 10 –phred33’. Potential host contamination was identified by mapping the trimmed reads against the GRCh38 human genome primary assembly from Ensemble (Cunningham et al., 2022) using *Bowtie2* v2.5.0 (Langmead & Salzberg, 2012). Reads showing mapping to the human genome were identified using *Samtools* v1.15.1 and filtered from fastq files using *seqkit* v2.3.0 (Shen et al., 2016). Paired end reads were analysed to identify amplicon sequence variants (ASVs) using *Quantitative Insights Into Microbial Ecology* (*QIIME2*) v2023.5.1 (Bolyen et al., 2019) and the *DADA2* v1.26.0 (Callahan et al., 2016) denoising package in R v4.2.3 (R Core Team, 2023). ASVs were assigned taxonomy using a classifier trained using the REference Sequence annotation and CuRatIon (RESCRIPt) module in *QIIME2* (Robeson et al., 2021). The classifier was trained using the SILVA v138.1 database (Quast et al., 2013), with sequences identified based on the 341F/805R primers used for PCR. Samples with fewer than 20,000 reads were removed from analysis. The ASV table was rarefied such that all samples had a sequence count equal to the sample with the lowest sequence depth of those passing filtering prior to analysis; main and sensitivity analyses of the primary analysis and main and sensitivity analysis of the sub‐analysis were each conducted at the same rarefied depth.

### 2.11 Blood analysis

Serum intestinal fatty acid binding protein (I‐FABP) concentration (DY990, R&D Systems, Minneapolis, MN, USA; dilution 1:5; intra‐assay CV 3.8%), plasma claudin 3 (CLDN‐3) concentration (NBP2‐75328, Novus Biologicals, Centennial, CO, USA; neat; intra‐assay CV 6.0%), plasma LPS binding protein (LBP) concentration (DY870‐05, R&D Systems; dilution 1:800; intra‐assay CV 4.1%), plasma soluble cluster of differentiation 14 (sCD‐14) concentration (DY870‐05, R&D Systems; dilution 1:800; intra‐assay CV 2.7%), and serum zonulin concentration (K5601, Immundiagnostik AG, Bensheim, Germany; dilution 1:20; intra‐assay CV 6.2%) were analysed in duplicate using commercially available ELISA kits and quantified using a plate reader (MRX, Dynex Technologies, Chantilly, VA, USA). Blood markers of serum creatine (Cr) and alanine transaminase (ALT) were provided, where possible, for severe EHI cases by the medical staff at CTCRM (Roche Cobas c 702, Roche Diagnostics, Basel, Switzerland).

### 2.12 Statistical analyses

Statistical analyses were conducted using R v4.3.1 (R Core Team, 2023). The null hypotheses were rejected where *P* < 0.05. Participant and loaded march characteristics, and blood biomarkers were tested for normality using the Shapiro–Wilk test. Normally distributed data are presented as means ± SD and between‐group differences assessed using an independent samples Student's *t*‐test; if the assumption of equal variances was violated, Welch's *t*‐test was used. If the assumption of normality was violated, data are presented as medians (IQR), and differences assessed using the Mann–Whitney *U*‐test. Effect sizes are reported as Cohen's *d* (*d* < 0.2: trivial; >0.2: small; >0.5: moderate; >0.8: large) (Cohen, 2013) and rank biserial correlation (*r* < 0.3: small; 0.3–0.5: medium; >0.5: large) (Cliff, 1993; Cohen, 2013; Kerby, 2014) for normally and non‐normally distributed data, respectively. Descriptives, normality and independent *t*‐tests were conducted using the *jamovi* v2.3.4 (Selker et al., 2022) package.

For GI microbiota analyses, α‐diversity was assessed using: (i) observed diversity; (ii) the Chao1 index (Chao, 1984); (iii) the Shannon index (Shannon, 1948); and (iv) the Simpson index (Simpson, 1949). Principal coordinates analysis (PCoA) plots were produced based on the Bray–Curtis dissimilarity measure (Bray & Curtis, 1957) to assess β‐diversity. Analyses of α‐ and β‐diversity was performed using the *phyloseq* package v1.44.0 (McMurdie & Holmes, 2013). The effect of group (non‐EHI *vs*. EHI) on bacterial β‐diversity was assessed using permutational multivariate analysis of variance (PERMANOVA) analysis with the *adonis2* function from the *vegan* v2.6‐4 package (Oksanen et al., 2022) using Bray–Curtis dissimilarity and 9999 permutations. The Firmicutes:Bacteroidota (F:B) ratio, and relative abundance of taxa at a phylum to genus taxonomic level were compared between groups. Comparisons of relative abundance were adjusted for multiple testing using the Benjamini and Hochberg (BH) correction (Benjamini & Hochberg, 1995). Differential abundance analysis was performed using the *DESeq2* v1.36.0 (Love et al., 2014), *ALDEx2* v1.34.0 (Fernandes et al., 2014), and *ANCOMBC* v2.4.0 packages (Lin & Peddada, 2020). A *P*‐value <0.05 was used to identify significantly different ASVs, and adjusted *P*‐values (using BH correction) were used for all three methods. For *DESeq2*, the non‐rarefied feature tables were passed to the *phyloseq_to_deseq2* function. Within the *DESeq* function, the Wald test was used and estimation of size factors set to ‘poscounts’ (Nearing et al., 2022). A fold change threshold of 2‐fold difference between the groups with an adjusted *P*‐value (adjusted for multiple testing using BH correction) was obtained. For *ALDEx2*, the non‐rarefied feature tables were passed to the *aldex* function which generated Monte Carlo samples of the Dirichlet distribution for each sample and converted each instance using a centred log‐ratio transform. Wilcoxon tests were performed on the transformed realisations, with the output returning the BH adjusted *P*‐values. For *ANCOMBC*, the non‐rarefied feature tables were passed to the *ancombc* function, with *P_adj_method* set to ‘BH’, to obtain adjusted *P*‐values. Counts of differentially abundant ASVs between all three packages were visualised with the *ggvenn* package v0.1.10 (Yan, 2023). Differentially abundant ASVs detected in all three methodologies were considered significant (Nearing et al., 2022).

In addition, to the main analysis, a sub‐group analysis was undertaken for severe EHIs and their pair matched non‐EHI controls only (i.e., excluding mild EHI cases and their pair matched non‐EHI control). Finally, to minimise potential confounding effects from reported NSAID use (Rogers & Aronoff, 2016) and GI obstructive disorders (Michielan & D'Incà, 2015) on GI microbiota analysis, and specific blood biomarkers (e.g., I‐FABP; Van Wijck et al., 2012), for each analysis an additional sensitivity analysis was undertaken (Thabane et al., 2013), excluding participants (and their pair matched non‐EHI control/case) who reported NSAID use in the 1 week prior to the loaded march, and/or GI obstructive disorders.

## 3 RESULTS

### 3.1 6.4‐mile loaded march characteristics

The median (IQR) exercise duration was 66 (1) min, with a mean ± SD WBGT recorded prior to commencing the exercise of 10.8 ± 3.0°C (range 6.0–15.4°C) WBGT.

### 3.2 Characteristics of EHI cases and matched non‐EHI controls

The participant characteristics of EHI cases and BMI‐ and fitness‐matched non‐EHI controls are presented in Table 1.

**TABLE 1:** Participant characteristics for individuals who did not develop an exertional heat illness (Non‐EHI: *n *= 79) and matched individuals who subsequently developed an exertional heat illness (EHI: *n *= 79).

Variable	Non‐EHI	EHI	*P*
**Anthropometry**			
Age (years)	22 (4)	21 (4)	0.903
Height (cm)	178.0 (7.9)	178.1 (8.0)	0.530
Body mass (kg)	81.2 ± 7.7	80.6 ± 7.8	0.669
Body mass index (kg^.^m^−2^)	25.3 ± 1.8	25.4 ± 2.0	0.786
Body surface area (m^2^)	1.99 (0.18)	1.97 (0.15)	0.613
Body surface area: mass ratio (cm^2^ kg^−1^)	247 ± 11	247 ± 11	0.944
**Fitness**			
RMFT (s)^a^	1506 ± 113	1554 ± 149	0.092
PT quartile rank^b^	2 (2)	3 (0)	0.273
BFT (s)^c^	240 ± 18	241 ± 23	0.954
**Temperature**			
Peak *T*_gi_ (°C)	38.9 (0.6)^d^	39.5 (0.8)^e^	< 0.001

Parametric data are presented as means ± SD; non‐parametric data are presented as medians (IQR).

^a^ *n *= 43 per group.

^b^ *n *= 6 per group.

^c^ *n *= 21 per group.

^d^ *n *= 60.

^e^ *n *= 75.

Abbreviations: BFT: bottom field test time; EHI: exertional heat illness, PT: physical trainer; RMFT: Royal Marines 2.5‐mile fitness test time; *T*_gi_: gastrointestinal temperature.

### 3.3 GI microbiota

Three samples were removed due to having fewer than 20,000 ASVs detected; their respective pair was also removed, leaving *n *= 152 (76 per group). Samples were rarefied to the minimum ASV depth across the remaining data set of 21,919 reads. For the additional sensitivity analysis, 13 participants reported NSAID use (one of the 13 also reported a GI disorder). These participants and their respective matched pair were removed from the additional sensitivity analysis (*n *= 26). Additionally, two samples were removed from the sensitivity analysis due to having fewer than 20,000 ASVs detected; their respective pair was also removed leaving a cohort total of *n *= 128 (64 per group) for sensitivity analysis. Samples for the sensitivity analysis were rarefied to the same depth as the main primary analysis.

**FIGURE 2:**
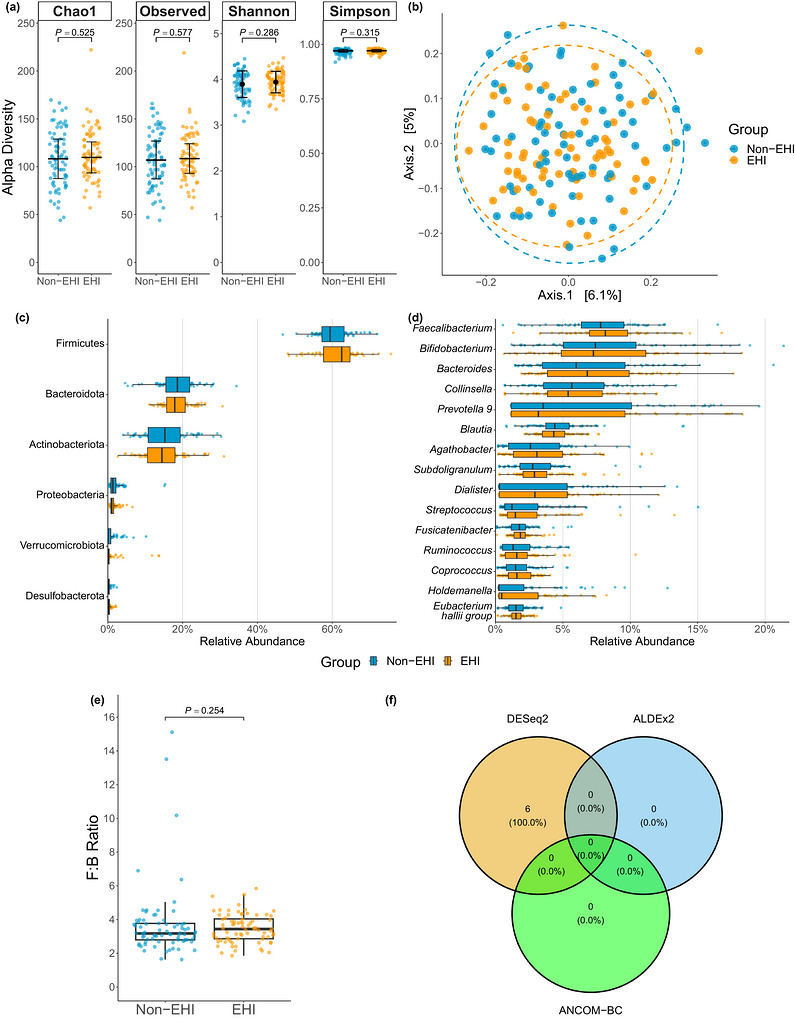
Comparison of gastrointestinal microbiota in individuals who did not develop an exertional heat illness (Non‐EHI: *n *= 76) and matched individuals who subsequently developed an exertional heat illness (EHI: *n *= 76). (a) α‐Diversity indices in non‐EHI controls and EHI cases; from left to right: Chao1, Observed, Shannon and Simpson (coloured dots represent individual data; large black dot represents mean ± SD; large wide bar represents median (interquartile range)). (b) Principal coordinate analysis (PCoA) based on Bray–Curtis dissimilarity measure to assess β‐diversity in non‐EHI controls and EHI cases. (c) Median (IQR) and individual relative abundance of 6 most abundant phyla in non‐EHI controls and EHI cases. (d) Median (IQR) and individual relative abundance of 15 most abundant genera in non‐EHI controls and EHI cases. (e) Median (IQR) and individual Firmicutes:Bacteroidota (F:B) ratio in non‐EHI controls and EHI cases. (f) Venn diagram comparing significant differentially abundant amplicon sequence variants between non‐EHI controls and EHI cases with three differential abundance methods (DESeq2, ALDEx2 and ANCOM‐BC).

Indices of bacterial α‐diversity were not significantly different between non‐EHI controls and EHI (Figure 2a). PERMANOVA identified no significant differences (*F*_(1,152)_ = 0.712, *P* = 0.993) in β‐diversity between non‐EHI controls and EHI (Figure 2b). The results of the sensitivity analysis excluding NSAID users supported the results of the overall cohort bacterial diversity analysis for both α‐diversity and β‐diversity (PERMANOVA: *F*_(1,126)_ = 0.800, *P* = 0.936). No significant between‐group differences were detected in the relative abundance of any identified taxa at the phylum level (top 6 phyla by relative abundance are presented in Figure 2c) or in the F:B ratio between non‐EHI controls and EHI (3.2 (1.0) vs. 3.4 (1.2), *P =* 0.254, *r* = 0.11; Figure 2e). No significant between‐group differences were detected in the relative abundance of any identified taxa at the genus level (the top 15 genera by relative abundance are presented in Figure 2d), nor indeed at any other taxonomic level that was assessed (i.e., class, order, family [data not shown]). The results of the sensitivity analysis supported the results of the microbial relative abundance analysis (F:B ratio: 3.2 (1.1) vs. 3.5 (1.2), *P =* 0.078, *r* = 0.18; no significantly different taxa at any taxonomic level).

DESeq2 analysis identified six significantly differentially abundant ASVs, whereas, both ALDEx2 and ANCOM‐BC identified no significantly differentially abundant ASVs (Figure 2f). Therefore, as there was no agreement for any ASVs across all three methods, it was concluded that there were no consistently differentially abundant ASVs between non‐EHI controls and EHI cases. The additional sensitivity analysis supported the results of the differential abundance analysis, with no significant ASVs identified across all three methods (number of significantly differentially abundant ASVs identified during sensitivity analysis: DESeq2: 9; ALDEx2: 0; ANCOM‐BC: 2).

### 3.4 Resting blood biomarkers

Blood samples were collected for 132 participants. We were unable to collect blood from three course cohorts (*n *= 24 participants) due to logistical reasons and could not obtain a blood sample from two participants. These two participants and their respective matched pair were removed from the analysis (*n *= 4). Lastly, we could not collect a plasma sample from one participant. This participant and their respective matched pair were also removed from analysis of EDTA samples only (*n *= 2). We therefore had a cohort total of *n *= 130 (65 per group) for serum analytes ([I‐FABP], [zonulin]), and *n *= 128 (64 per group) for plasma analytes ([CLDN‐3], [LBP], [sCD14]). For the additional sensitivity analysis, nine participants reported NSAID use (one of the nine also reported a GI disorder). These participants and their respective matched pair were removed from analysis (*n *= 18). Our sensitivity analysis therefore consisted of a cohort total of *n *= 112 (56 per group) for serum analytes, and *n *= 110 (55 per group) for plasma analytes.

**FIGURE 3:**
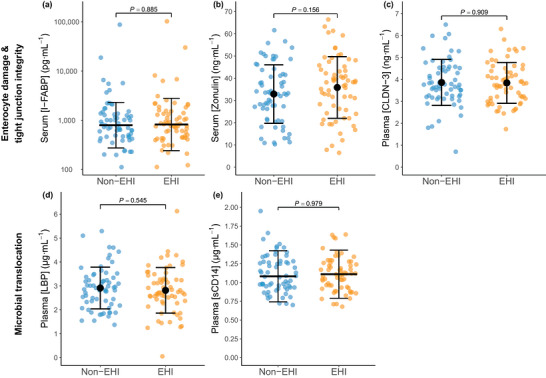
Comparison of baseline biomarkers of gastrointestinal barrier function in individuals who did not develop an exertional heat illness (Non‐EHI) and matched individuals who subsequently developed an exertional heat illness (EHI). (a) Serum [I‐FABP] (log scale), (b) serum [Zonulin], (c) plasma [CLDN‐3], (d) plasma [LBP], and (e) plasma [sCD14]. Coloured dots represent individual data; large black dot represents mean ± SD; large wide bar represents median (interquartile range). *n *= 65 per group for serum biomarkers; *n *= 64 per group for plasma biomarkers. CLDN‐3: claudin 3; I‐FABP: intestinal fatty acid binding protein; LBP: liposaccharide binding protein; sCD14: soluble Cluster of Differentiation 14.

None of the baseline biomarkers of GI barrier function significantly differed between non‐EHI controls and EHI cases (Figure 3). The results of the sensitivity analysis supported the results of the overall biomarker analysis.

### 3.5 Sub‐group analysis

As part of the sub‐group analysis, severe EHI cases (*n *= 24) were compared against their respective pair matched non‐EHI controls (i.e., excluding mild EHI cases and their controls). Severe EHI cases (*n *= 24) had a significantly greater peak *T*_gi_ than mild (*n *= 51) EHI cases (39.8 (0.7) vs. 39.2 (0.7)°C, *P* ≤ 0.001), and pair matched non‐EHI controls (see Table 2). The participant characteristics for the sub‐analysis are presented in Table 2. For the subset of severe EHI cases referred for serial blood sampling (*n* = 6), 100% met the criteria for end‐organ damage.

**TABLE 2:** Participant characteristics of individuals who did not develop an exertional heat illness (Non‐EHI: *n *= 24) and matched individuals who subsequently developed a severe exertional heat illness (Severe EHI: *n *= 24).

Variable	Non‐EHI	Severe EHI	*P*
**Anthropometry**			
Age (years)	22 (4)	22 (5)	0.708
Height (cm)	178.1 ± 4.6	179.3 ± 6.1	0.441
Body mass (kg)	80.4 ± 7.4	82.1 ± 8.2	0.463
BMI (kg m^−2^)	25.3 ± 1.8	25.5 ± 2.3	0.728
Body surface area (m^2^)	1.98 ± 0.11	2.01 ± 0.12	0.410
Body surface area: mass ratio (cm^2^ kg^−1^)	248 ± 11	246 ± 11	0.632
**Fitness times**			
RMFT (s)^a^	1483 (149)	1564 (94)	0.157
RMFT PT quartile rank^b^	2 (1)	3 (0)	0.059
BFT (s)^c^	243 ± 34	244 ± 36	0.958
**Temperature**			
Peak *T*_gi_ (°C)	38.9 (0.2)^d^	39.8 (0.7)^e^	< 0.001

Parametric data are presented as means ± SD; non‐parametric data are presented as medians (IQR).

^a^ *n *= 12 per group.

^b^ *n *= 3 per group.

^c^ *n *= 5 per group.

^d^ *n *= 16. ^e^ *n *= 24.

Abbreviations: BFT: bottom field test time; EHI: exertional heat illness; PT: physical trainer, RMFT: Royal Marines 2.5‐mile fitness test time, *T*_gi_: gastrointestinal temperature.

#### 3.5.1 GI microbiota

Sub‐group analysis samples were rarefied to the minimum ASV depth of 22,061 reads. For sensitivity analysis, four individuals reported NSAID use. These individuals and their respective matched pair were removed from analysis (*n *= 8), resulting in *n *= 40 (20 per group) remaining for microbiota analysis. Sensitivity analysis samples were rarefied to the same depth as the main sub‐group analysis.

**FIGURE 4:**
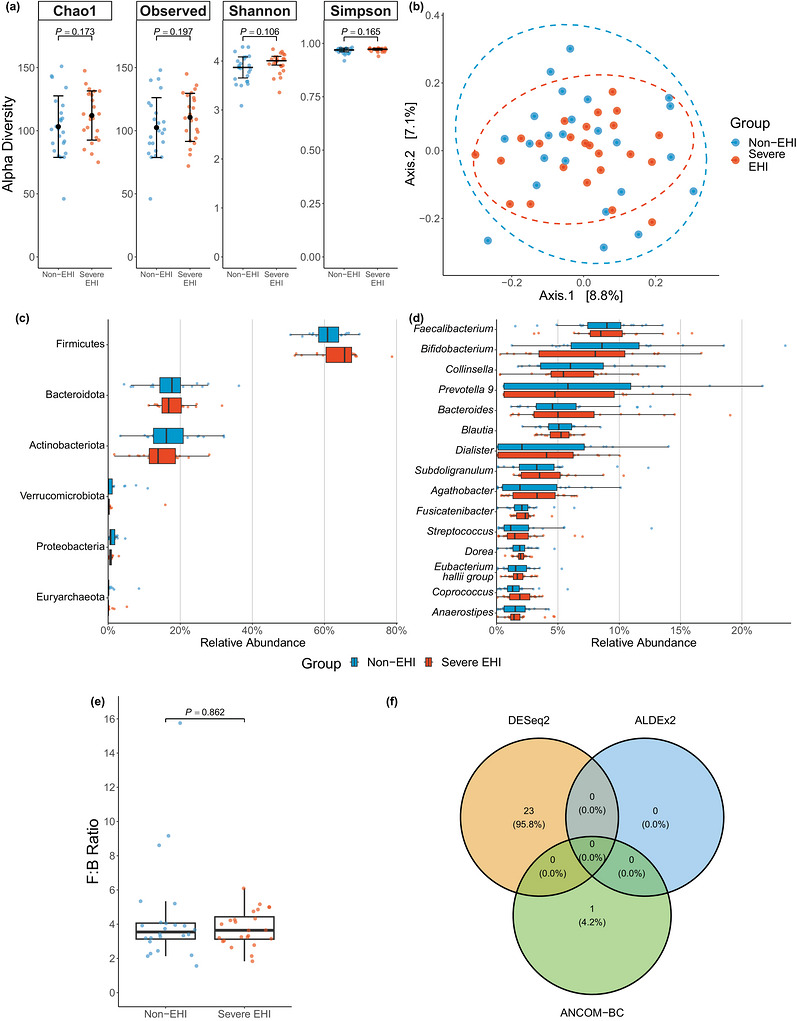
Comparison of gastrointestinal microbiota in individuals who did not develop an exertional heat illness (Non‐EHI: *n *= 24) and matched individuals who subsequently developed a severe exertional heat illness (Severe EHI: *n *= 24). (a) α‐Diversity indices in non‐EHI controls and EHI cases; from left to right: Chao1, Observed, Shannon and Simpson (coloured dots represent individual data; large black dot represents mean ± SD; large wide bar represents median (interquartile range)). (b) Principal coordinate analysis (PCoA) based on Bray–Curtis dissimilarity measure to assess β‐diversity in non‐EHI controls and EHI cases. (c) Median (IQR) and individual relative abundance of 6 most abundant phyla in non‐EHI controls and EHI cases. (d) Median (IQR) and individual relative abundance of 15 most abundant genera in non‐EHI controls and EHI cases. (e) Median (IQR) and individual Firmicutes:Bacteroidota (F:B) ratio in non‐EHI controls and EHI cases. (f) Venn diagram comparing significant differentially abundant amplicon sequence variants between non‐EHI controls and EHI cases with three differential abundance methods (DESeq2, ALDEx2 and ANCOM‐BC). ns: not significant, *P *> 0.05.

Indices of α‐diversity were not significantly different between non‐EHI controls and severe EHI cases (Figure 4a). PERMANOVA identified no significant differences (*F*_(1,46)_ = 0.929, *P* = 0.685) in β‐diversity between non‐EHI controls and severe EHI cases (Figure 4b). The results of the sensitivity analysis supported the results of the sub‐group bacterial diversity analysis for both α‐ and β‐diversity (PERMANOVA: *F*_(1,38)_ = 0.865, *P* = 0.844). No significant between‐group differences were detected in the relative abundance of any taxa at the phylum level (top 6 phyla are presented in Figure 4c) or in the F:B ratio between non‐EHI controls and severe EHI (3.6 (0.9) vs. 3.6 (1.3), *P =* 0.862, *r* = 0.03; Figure 4e). No significant between‐group differences were detected in the relative abundance of any taxa at the genus level (the top 15 genera are presented in Figure 4d), nor at any other taxonomic level (i.e., class, order, family [data not shown]). The results of the sensitivity analysis supported the results of the bacterial relative abundance analysis (F:B ratio: 3.5 (1.2) vs. 3.6 (1.3), *P =* 0.547, *r* = 0.12 with no significantly different abundant taxa at any taxonomic level).

DESeq2 and ANCOM‐BC analysis identified 23 and 1 significantly differentially abundant ASV(s), respectively, whereas ALDEx2 identified no significantly differentially abundant ASVs. (Figure 4f). Therefore, as there was no agreement for any ASVs across all three methods, we concluded that there were no differentially abundant ASVs between non‐EHI controls and severe EHI cases. The sensitivity analysis supported the results of the differential abundance analysis, that there were no consistent significant changes in abundance between EHI and non‐EHI controls (number of significantly differentially abundant ASVs identified during sensitivity analysis: DESeq2: 27; ALDEx2: 0; ANCOM‐BC: 66).

#### 3.5.2 Markers of GI barrier integrity

We were unable to collect blood from three course cohorts (*n *= 8) due to logistical reasons, and unable to collect blood from two participants. These two participants and their respective matched pair were removed from analysis (*n *= 4). We therefore had a total of *n *= 36 (18 per group). For sensitivity analysis, two participants reported NSAID use; these participants and their respective matched pair were removed from analysis (*n *= 4). We therefore had a total of *n *= 32 (16 per group) for the sensitivity analysis.

**FIGURE 5:**
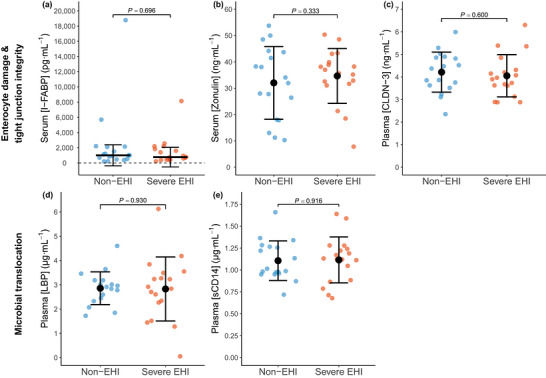
Comparison of baseline (1 day prior to the 6.4‐mile loaded march) biomarkers of gastrointestinal barrier function in individuals who did not develop an exertional heat illness (Non‐EHI: *n *= 18) and matched individuals who subsequently developed a severe exertional heat illness (Severe EHI: *n *= 18). (a) Serum [I‐FABP], (b) serum [zonulin], (c) plasma [CLDN‐3], (d) plasma [LBP], and (e) plasma [sCD14]. Coloured dots represent individual data; large black dot represents mean ± SD, large wide bar represents median (interquartile range). CLDN‐3: claudin 3; I‐FABP: intestinal fatty acid binding protein; LBP: liposaccharide binding protein; sCD14: soluble cluster of differentiation 14.

Baseline biomarkers of GI barrier function did not significantly differ between non‐EHI controls and severe EHI cases (Figure 5). The results of the sensitivity analysis supported the results of the overall biomarker analysis.

## 4 DISCUSSION

Using a novel *prospective* study design we have, for the first time, examined the GI microbiota composition and indices of GI barrier integrity in individuals who subsequently (within 3 days) developed an EHI, and made comparison to matched non‐EHI control participants who did not develop an EHI during the same activity. The main findings of this study were that neither the GI bacterial diversity and relative abundance nor indices of GI barrier integrity differed between individuals who subsequently developed an EHI and matched non‐EHI controls. These findings persisted when undertaking an additional sub‐analysis of severe EHI cases only, and sensitivity analyses excluding individuals who reported prior NSAID use and/or GI disorder. We therefore reject our experimental hypothesis.

Although it has been suggested that the composition of an individual's gut microbiota may be implicated in the aetiology of EHI (Armstrong et al., 2018; Roberts et al., 2021), relevant empirical data were lacking until the present study. We recently demonstrated that, except for the Simpson α‐diversity index, the composition of the GI microbiota of individuals who have suffered a previous EHI (∼4 months post‐EHI) and matched controls without prior EHI history was not different (Gould et al., 2025). These data suggested that the GI microbiota composition does not contribute to previously reported high EHI reoccurrence rates (Nelson et al., 2018a; Stearns et al., 2020), and is not substantially impacted by the initial EHI episode (or recovers within ∼4 months). However, because the GI microbiota composition may show temporal changes for multifarious reasons (Baümler & Sperandio, 2016; David et al., 2014; Dethlefsen et al., 2008; Hou et al., 2022; Mohr et al., 2020; Monda et al., 2017; Rogers & Aronoff, 2016; Rooks & Garrett, 2016), the retrospective nature of our previous study did not preclude that the composition of the GI microbiota and associated effects on GI barrier integrity and inflammation may have contributed to the initial EHI episode.

To address this gap in understanding, the present study assessed the GI microbiota and indices of GI barrier integrity in close proximity *prior to* the development of an EHI. In addition, we employed rigorous control matching to account for potential confounding factors on the GI microbiota, with each EHI case matched to a non‐EHI control participant from within the same course cohort living in the same accommodation, with similar dietary provision, undertaking the same daily physical activity as part of their training course, and undertaking the same 6.4‐mile loaded march. This unique design effectively controlled for potential confounders including: diet (Singh et al., 2017), seasonal differences (Gacesa et al., 2022), physical activity levels (Monda et al., 2017) and differences in ambient conditions that could affect physical performance (Jenkins et al., 2023). We additionally matched our non‐EHI controls to our EHI cases for BMI (Vujkovic‐Cvijin et al., 2020) and cardiorespiratory fitness (Estaki et al., 2016). With this prospective design, and strong experimental controls, we demonstrate that there were no differences between EHI cases and non‐EHI controls for (i) α‐diversity indices, (ii) β‐diversity, (iii) relative abundance of detected microbial taxa from a phylum to genus taxonomic level, and (iv) differential abundance of identified ASVs.

Given that the GI system has been proposed to be implicated in the aetiology of the more severe forms of EHI (Garcia et al., 2022; Lim, 2018; Ogden et al., 2020; Roberts et al., 2021), we also undertook a sub‐analysis of GI microbiota composition excluding the mild EHI cases. Importantly, our severe EHI cases exhibited CNS dysfunction, a key discriminatory feature of EHS (Laitano et al., 2019), as well as a significantly higher peak deep body temperatures than our mild EHI cases (severe EHI, 39.8 (0.7)°C vs. mild EHI, 39.2 (0.7)°C; *P* ≤ 0.001). The results of these sub‐analyses were consistent with the overall analyses and again indicated that there were no significant differences in the composition of the GI microbiota between severe EHI cases and their pair matched non‐EHI control. However, our power to detect this sub‐analysis would have been affected by our smaller sample size, and the effect sizes for the α‐diversity indices tended to be larger for this sub‐analysis than the overall analysis. Nevertheless, all of the effect sizes remained ‘small’ and the measures of central tendency indicated a numerically *greater* α‐diversity in the severe EHI cases than in the matched non‐EHI controls, which is typically associated with superior GI health (Pickard et al., 2017).

Due to the potential influence of NSAIDs on the GI microbiota (Rogers & Aronoff, 2016), we undertook an additional sensitivity analysis where we removed NSAID users from our analysis of all EHI cases, as well as from our sub‐analysis of severe EHI cases only. In each instance the difference between groups remained non‐significant and the effect sizes remained small, suggesting that the reported NSAID usage had little impact on the GI microbiota composition. Taken together the consistent findings reported across each of our analyses (i.e., main analysis, sub‐analysis and sensitivity analysis) suggest that, when relevant confounders are effectively controlled for, the composition of the GI microbiota does not contribute to EHI risk during a loaded march exercise.

Increased permeability of the GI epithelial barrier and downstream effects on inflammation have been highlighted in the aetiology of a number of chronic inflammatory diseases (Camilleri, 2019; Farhadi et al., 2003; Fasano, 2020) with similar pathophysiology to some aspects of EHI aetiology (Garcia et al., 2022; Lim, 2018; Ogden et al., 2020). Accordingly, in addition to assessing the composition of the GI microbiota, we also assessed baseline biomarkers of tight junction integrity (serum [zonulin]; plasma [CLDN‐3]), enterocyte damage (serum [I‐FABP]) and microbial translocation (plasma [LBP]; plasma [sCD14]) (Ogden et al., 2020). Previous research has shown that some of these biomarkers are substantially elevated in blood samples obtained after an individual has suffered an EHI episode (Walter et al., 2021), but this type of post‐EHI assessment does not provide insight into whether GI epithelial barrier dysfunction was a *cause* or a *consequence* of the EHI episode. Our prospective research approach, in which these biomarkers were assessed from a blood sample obtained 1 day *prior* to the EHI event, demonstrated that there was no difference in any of the measured biomarkers of GI barrier integrity between individuals who subsequently develop an EHI and matched non‐EHI controls who did not develop an EHI. These findings persisted with our sub‐analysis of severe EHI cases, as well as with our sensitivity analysis in which individuals reporting NSAID usage or GI disorders were excluded. Accordingly, our data indicate that pre‐existing impairments in tight junction integrity, enterocyte damage or microbial translocation are not causally linked to the subsequent EHI event, and where pronounced changes in these biomarkers are evident post‐EHI, they are likely to occur as a consequence of the activity (Ogden et al., 2020). Interestingly, large inter‐individual variation in serum [I‐FABP] was observed, with two individuals in each group (none of whom reported NSAID usage) having values approaching, or exceeding, those reported following an EHI (15,389 ± 8547 pg mL^−1^; Walter et al., 2021) and further indicating that enterocyte damage may not be causally linked to EHI development.

The findings of the present study must be interpreted within the context of the experimental approach employed. The 6.4‐mile loaded march is a fixed‐pace activity which may bias towards greater EHI risk in those with lower fitness. Whilst we have controlled for this effect as far as possible within our control‐matching process, which incorporated an index of participant fitness, we cannot exclude the possibility that our conclusions would have differed during self‐paced exercise. Likewise, caution should be taken in extrapolating our findings to women; however, the study design provides high ecological validity for military personnel undertaking arduous training in realistic operational conditions. Our rigorous controls and pair‐matching are a notable strength supporting investigation of the independent influence of aspects of the GI system on EHI aetiology, but the potential confounding factors that we have controlled for could influence EHI risk through secondary effects on the GI microbiota and GI barrier integrity. However, our sensitivity analysis excluding NSAID users and GI disorders suggests that this was not the case for these factors. We recorded ambient temperature in a fixed location prior to commencing the exercise and cannot preclude that it may have changed during the activity, but any changes would have been experienced similarly for each EHI case and their matched control within their course cohort. Similarly, we cannot preclude that our findings would have differed under more thermally stressful environments or during different activity (e.g., longer duration and/or more intense).

The EHI classifications that we employed were derived from key discriminatory features of EHI as outlined in expert consensus statements (Armstrong et al., 2007; Casa et al., 2015; Hemingway et al., 2025; Laitano et al., 2019; Roberts et al., 2021) and are consistent with the assertion that CNS dysfunction should have primacy with EHI diagnosis and a classification. A notable strength of the present study is that we used the validated QCS to objectively assess CNS disturbance in our participants (Irons et al., 2002), and whilst it was not possible to obtain blood samples in all of our severe EHI cases, in every instance where such samples were obtained, we found evidence of end‐organ damage. We also acknowledge that serum creatinine may increase after exercise due to increased release from muscle cells (Beunders et al., 2020), but given the magnitude of increase in serum creatinine typically seen with strenuous endurance exercise (Hodgson et al., 2017) compared to the post exercise values seen in our participants (e.g., 148.2 ± 17.3 µmol L^−1^on the day of the EHI), it is likely that end‐organ damage was present in our severe EHI cases. It should also be noted that three of our non‐EHI control participants reported a QCS score ≤11 but were missing the *T*_gi_ or blood testing data for definitive EHI classification. However, further analysis with the removal of these individuals and their matched pairs did not change our conclusions.

Finally, our GI microbiota analyses were conducted on extractions from stool samples to represent the GI tract. This approach can differ in sensitivity from invasive procedures such as biopsies but also has fewer negative factors with reduced risk to the individual (Zhou et al., 2021). Although this approach does not enable characterisation of site‐specific differences in microbiota along the GI tract (Shalon et al., 2023), overall bacterial community differences can be safely, clearly and rapidly observed using this methodology. The bacterial community analysis provided by the 16S rRNA gene sequencing enables rapid comparison of different individuals and cohorts in multi‐factor studies. However, to obtain more detailed taxonomic and functional insights, other approaches such as whole metagenomic sequencing (Knight et al., 2018) and transcriptomics should be employed in future analyses. Furthermore, future analyses could include all components of the microbiota, including the virome and mycobiome. In addition, we have studied a highly homogeneous cohort (in terms of sex, age, fitness, diet, activity level, etc.), which would reduce variability relative to the broader population. Given the high reported rates of EHI in our cohort, we cannot exclude the possibility that this group possesses an enterotype that is more conducive to EHI development than that occurring in the broader population.

In conclusion, using a unique prospective study design with rigorous controls, we have shown, for the first time, that the GI microbiota and indices of GI barrier integrity do not differ between individuals who subsequently develop an EHI during exercise and pair matched non‐EHI controls, completing the same task, on the same day, who did not develop an EHI. These findings persisted when conducting an additional sub‐group analysis consisting of only severe EHI cases, and additional sensitivity analysis excluding individuals who reported NSAID use and GI disorders. Together, this evidence indicates that for this cohort and experimental model, when potential confounding variables are controlled for, neither the composition of the GI microbiota nor baseline indices of GI barrier integrity contribute to a heightened risk of developing an EHI.

### 4.1 Translational perspective

We prospectively examined if the gut microbiota composition and markers of gut barrier integrity differed amongst individuals who subsequently developed an exertional heat illness (EHI) during strenuous exercise and matched control participants who did not develop an EHI during the same activity. There were no significant differences in the gut microbiota composition, nor in markers of gut barrier integrity between EHI cases and matched controls. As such, these measures do not appear to increase susceptibility to EHI. However, future research is required to examine if these findings translate to longer duration, or self‐paced exercise, as well as other cohorts.

## AUTHOR CONTRIBUTIONS

Alex A. M. Gould, Neil P. Walsh, Michael J. Tipton, Michael J. Zurawlew, Samuel C. Robson, Janis K. Shute, Joy E. M. Watts, Hayley C. Tyson, Megan R. Robinson, Andrew J. Roberts, Alex J. Rawcliffe, Ross Hemingway, Jo Corbett conceived and designed research; Alex A. M. Gould, Michael J. Zurawlew, Hayley Tyson, Megan Robinson performed experiments; Alex A. M. Gould, Samuel C. Robson, Janis K. Shute analysed data; Alex A. M. Gould, Neil P. Walsh, Michael J. Tipton, Michael J. Zurawlew, Samuel C. Robson, Janis K. Shute, Joy E. M. Watts, Hayley Tyson, Megan R. Robinson, Andrew J. Roberts, Alex J. Rawcliffe, Ross Hemingway, Jo Corbett interpreted the results of the experiments; Alex A. M. Gould, Samuel C. Robson prepared figures; Alex A. M. Gould, Samuel C. Robson, Joy E. M. Watts, Jo Corbett drafted the manuscript; Alex A. M. Gould, Neil P. Walsh, Michael J. Tipton, Michael J. Zurawlew, Samuel C. Robson, Janis K. Shute, Joy E. M. Watts, Hayley Tyson, Megan R. Robinson, Andrew J. Roberts, Alex J. Rawcliffe, Ross Hemingway, Jo Corbett edited and revised the manuscript. All authors have read and approved the final version of this manuscript and agree to be accountable for all aspects of the work in ensuring that questions related to the accuracy or integrity of any part of the work are appropriately investigated and resolved. All persons designated as authors qualify for authorship, and all those who qualify for authorship are listed.

## CONFLICT OF INTEREST

None declared.

## Data Availability

The data that support the findings of this study are available from the corresponding author upon reasonable request and subject to approval from the UK Ministry of Defence. The code used for the analysis of data in this manuscript is available under a GNU General Public License V3.0 at https://github.com/AlexG0uld/2024_Prospective_EHI_GI_Paradigm.
